# Evaluation of antioxidant–surfactant interactions using size distribution Taylor dispersion analysis: measuring antioxidant partitioning and size of native-state micelles

**DOI:** 10.1016/j.crfs.2025.101142

**Published:** 2025-07-11

**Authors:** Erwann Durand, Théo Troncho, Pierre Villeneuve

**Affiliations:** a CIRAD, UMR QualiSud, F-34398, Montpellier, France; b QUALISUD, Univ Montpellier, Avignon Université, CIRAD, Institut Agro, IRD, Université de La Réunion, Montpellier, France

**Keywords:** Antioxidant, α-Tocopherol, Curcumin, Micelles, Taylor dispersion analysis

## Abstract

In food emulsions, excess surfactants often lead to the formation of micelles, which play a critical role in modulating the localization and efficacy of antioxidants. The interactions between antioxidants and micelles can either enhance or impair antioxidant function. Despite this importance, methodologies to study these interactions in native-state micelles remain limited. In this study, we employed Size Distribution - Taylor Dispersion Analysis (SD-TDA), a fast and absolute technique for measuring diffusion coefficients and thus hydrodynamic radii, to investigate antioxidant–surfactant interactions in sodium dodecyl sulfate (SDS) micelles, both below and above its critical micelle concentration. Studied antioxidants include α-tocopherol, curcumin, gallic acid, propyl gallate, and octyl gallate. The results revealed distinct behaviors: while gallic acid remained in the aqueous phase regardless of SDS presence, octyl gallate appeared to co-micellize with SDS. Curcumin was fully incorporated into SDS micelles, with micelle size increasing from 4.68 ± 1.52 nm to 6.92 ± 0.79 nm at the maximum loading capacity of 3.57 ± 0.05 mM curcumin with 20 mM SDS. α-Tocopherol also localized within SDS micelles; however, above 2.5 mM, three distinct populations were identified using SD-TDA—free tocopherol in water, tocopherol in small micelles, and swollen SDS micelles. Unlike Dynamic Light Scattering, SD-TDA successfully resolved these populations and provided quantitative insights into their size and α-tocopherol partitioning. These findings highlight the potential of SD-TDA as a powerful tool for studying antioxidant partitioning and micellar interactions, opening the way for more in-depth investigations into the role of micelles in antioxidant efficiency in emulsified systems.

## Highlights

• SD-TDA revealed distinct antioxidant partitioning in SDS micellar systems.
• Gallic acid stayed in water; propyl gallate showed partial micellar binding.
• G8 and curcumin fully entered micelles, enlarging size with concentration.
• α-Tocopherol formed multiple populations at higher concentrations.
• Unlike DLS, SD-TDA provided high-resolution distinction of coexisting populations.

## Graphical abstract

**Figure:**
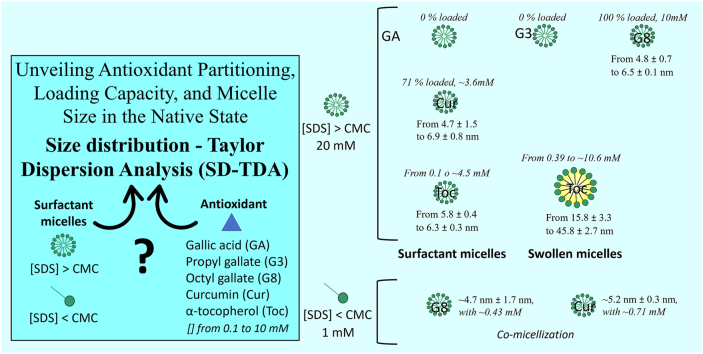

## 1 Introduction

Lipid oxidation is a major concern in the food industry, as it can degrade unsaturated fatty acids, leading to off-flavor development, loss of nutritional quality, and the formation of oxidation products that are suspected to be harmful to human health (Domínguez et al., 2019; Barden and Decker, 2013; German, 1999). Lipid oxidation can take place through enzymatic oxidation by lipoxygenases, photooxidation via the activation of a sensitizer by light, or autooxidation. This phenomenon is influenced by various factors such as temperature, light exposure, oxygen levels, lipid unsaturation, and the presence of pro-oxidant compounds like photosensitizers, metals, and radical species (Schaich, 2020; Durand et al., 2025; Hennebelle et al., 2024). Additionally, the food product's composition, structure, and properties characteristics affect its susceptibility to lipid oxidation. Factors such as moisture content, water activity, structural arrangement, rheological properties, and physical state all play a role. While lipid oxidation mechanisms have been extensively investigated in bulk oils, the majority of dietary lipids are consumed in the form of emulsions, particularly oil-in-water (O/W) emulsions that are commonly found in a wide range of products, including beverages, milk, infant formulas, dairy products, mayonnaise or dressings. These emulsions are composed of oil droplets dispersed in a continuous aqueous phase, stabilized by surface-active molecules that accumulate at the oil-water interface. This interface is critical, as it serves as the contact point where lipids, oxygen, and pro-oxidants interact. The extensive interfacial area in emulsions increases lipid exposure to pro-oxidant agents, accelerating oxidation compared to bulk oils (McClements and Decker, 2000). Various factors influence lipid oxidation pathways, including droplet size, interfacial composition, emulsifier type, and the partitioning and reactivity of all substances involved. In emulsified systems, a common strategy to prevent lipid oxidation is the addition of antioxidants. These compounds differ in their chemical properties and mechanisms of action, which may include radical scavenging, metal chelation, singlet oxygen quenching, and inhibition of lipoxygenase activity. However, the efficiency of a selected antioxidant in emulsions is not only governed by its chemical reactivity but also by its physico-chemical behavior in particular its partitioning, mobility and diffusion in the system (Villeneuve et al., 2023). These physico-chemical properties of antioxidants are dependent on the type of antioxidants (structure, polarity, tension-actives properties) as well as their interactions with surfactant micelles that are present in the emulsion. Indeed, micelles play a crucial role in lipid oxidation within emulsified systems as carriers for antioxidants, pro-oxidants, and oxidation products, which are expected to significantly influence the rate of lipid oxidation. Various studies underlined the impact of antioxidant-micelles interactions on the oxidative stability of emulsions. For example, Huang et al. (1997) suggested that micellization of lipophilic antioxidants (methyl carnosate, carnosol, carnosic acid, α-tocopherol) in Tween 20 micelles could help to locate them at the oil/water interface and therefore, boost their antioxidant efficiencies (Huang et al., 1997). Similarly, Kiralan et al. (2014) showed that Tween 20 could solubilize tocopherols in the continuous phase of an emulsion by forming surfactant–tocopherol comicelles (Kiralan et al., 2014). These structures may act as a reservoir of antioxidants, enabling the replacement of oxidized antioxidants at the interface after they are consumed by free radicals. Inchingolo et al. (2021) investigated how SDS micelles influence the activity of α-tocopherol (Inchingolo et al., 2021). When α-tocopherol was introduced after emulsification at SDS concentrations exceeding 4 mM, a significant increase in its presence was detected in the aqueous phase. However, this solubilization occurred below the critical micelle concentration (CMC) of SDS (8.3 mM), suggesting that SDS and α-tocopherol formed comicelles at a lower CMC than that of pure SDS. Additionally, their findings indicated that α-tocopherol's ability to reduce metals - linked to its prooxidant activity - was likely diminished when incorporated into a micelle. Stöckmann, Schwarz, and Huynh-Ba (2000) examined the distribution of gallic acid and its esterified derivatives (gallate esters) in emulsions stabilized by different surfactants, including SDS, CTAB, and Brij 58. In a 1 % SDS emulsion (above its CMC), it was found that the solubilization of gallate esters into the oil phase or SDS increased with alkyl chain length, whereas gallic acid was solubilized exclusively in the aqueous phase and not in SDS or the lipid phase (Stöckmann et al., 2000). Using ethyl gallate as a reference antioxidant, the study revealed that CTAB had the highest solubilization capacity, followed by Brij 58 and SDS. The authors suggested that these variations in solubilization could be linked to differences in the hydrogen bond basicity of the surfactants, which depends on their negative counter-ions. The study further explored the antioxidant activity of gallic acid and ethyl gallate in a stripped corn oil-in-water emulsion. While gallic acid exhibited no antioxidant effect, ethyl gallate was most effective in SDS-stabilized emulsions, followed by Brij 58, but had no impact in CTAB-stabilized systems. Interestingly, this order was the reverse of the surfactants' solubilization capacities. Since solubilization was attributed to hydrogen bonding, the researchers concluded that strong hydrogen bonding between antioxidants and emulsifiers could weaken the antioxidant effectiveness of the phenolic compound. With Nuclear Magnetic Resonance (NMR) and Electron Spin Resonance (ESR), Heins, McPhail et al. (2007) evaluated the activity of gallate antioxidants toward radicals depending on their location in micelles (Heins et al., 2007a). To investigate this, they used the lipophilic galvinoxyl radical, which localizes in the deeper hydrophobic regions of Brij 58, CTAB, and SDS micelles, along with the hydrophilic Fremy's radical, which dissolves exclusively in the aqueous phase of the SDS micellar solution and distributes between the aqueous phase and the headgroup region of Brij 58. With methyl, propyl, butyl, and octyl gallates, the authors observed a stoichiometric interaction between these antioxidants and galvinoxyl radicals in CTAB micelles, indicating that both were situated in the same region within the micelles. In contrast, in SDS micelles, the stoichiometry of gallates showed weak interactions with hydrophobic radicals, suggesting that SDS acted as a physical barrier, with the gallates positioned in the Stern layer of micelles. For Brij 58 micelles, the stoichiometry increased with antioxidant chain length, suggesting that the antioxidants were primarily located in the headgroup region. The enhanced lipophilicity associated with longer alkyl chains appeared to promote their partitioning into the micelle's palisade layer. The opposite trend was observed with hydrophilic Fremy's radicals, as the stoichiometry of gallates with these radicals decreased with increasing antioxidant chain length. Octyl gallate exhibited the weakest stoichiometry due to its deeper penetration into the polyoxyethylene region of Brij 58 micelles. Similar results were found with SDS micelles, where longer antioxidant chains were positioned deeper within the Stern layer, reducing their interaction with Fremy's radical, which is repelled by the anionic nature of SDS. More recently, Wang et al. (2024) used NMR (^1^H and NOESY) to explore the interaction between tocopherols and Tween 20 surfactant micelles (Wang et al., 2024). They observed that β-/γ-tocopherols were located near the ester group between the hydrophilic headgroup and the lauric acid tail group. As the concentration of Tween 20 increased, the chemical environment and interaction behavior of solubilized β-/γ-tocopherols gradually changed, with the tocopherol molecules increasingly solubilizing within Tween 20 micelles.

The studies outlined above highlight that (i) the interaction between micelles and antioxidants is multifactorial, depending on both the nature and concentration of the antioxidants and surfactants, and (ii) molecular interactions between antioxidants and surfactant micelles play a crucial role in modulating their efficacy and influencing the oxidative stability of oil-in-water emulsions. However, the literature on these phenomena remains limited, lacking in-depth studies and methodologies to thoroughly investigate the physicochemical interactions between antioxidants and surfactants in their native micellar form. Therefore, in the present work, we aim to investigate the potential of applying Size Distribution - Taylor Dispersion Analysis (SD-TDA) to measure the size and investigate interaction of antioxidants in native-state micelles. SD-TDA is a technique for measuring the diffusion coefficients and hydrodynamic radii of molecules. This rapid and absolute method relies on the dispersion of a solute plug as it moves through a uniform cylindrical tube under laminar Poiseuille flow (Hawe et al., 2011; Balog et al., 2017; Taylor, 1953). It involves injecting a solute plug into a flowing solvent within an open tubular column, where dispersion occurs due to a combination of radial diffusion and cross-sectional velocity variations. This technique enables accurate determination of diffusion coefficients and particle sizes in solution by utilizing the principles of Taylor dispersion and employing absorbance detection to track the sample's movement through a capillary. This method can be applied to analytes of nearly any size, from angstrom-scale to sub-micrometer, with significantly less interference from large particles or aggregates compared to other technique such as dynamic light scattering (DLS) (Moser and Baker, 2021). To date, SD-TDA has been proved to be a relevant method to evaluate the size of various nano objects such as single molecules, peptides and proteins aggregates (Norrild et al., 2024; Roca et al., 2024a, 2024b; Am et al., 2024), liposomes (Franzen et al., 2011), colloidal nanoparticles (Fortunatus et al., 2025; Malburet et al., 2022a, 2022b, 2023) or extracellular vesicles (Obeid et al., 2023). Given the limited literature and the inherent complexity of thoroughly investigating physicochemical interactions between antioxidants and surfactants in their native-state micellar form, this study aims to explore the potential of Size Distribution–Taylor Dispersion Analysis (SD-TDA) as a method to measure micelle size and probe antioxidant–micelle interactions (Roca et al., 2024b). In this study, SD-TDA was used to evaluate how the nature, concentration, and antioxidant-to-surfactant ratio of antioxidants such as α-tocopherol, curcumin, gallic acid, propyl gallate, and octyl gallate influence their interaction with surfactant molecules. SDS, selected as the surfactant, was tested both below and above its CMC in the presence of different antioxidants. A comparison between SD-TDA and DLS was conducted to highlight how SD-TDA can complement DLS, offering deeper insights into size measurements and antioxidant partitioning, particularly when multiple populations coexist.

## 2 Materials and methods

### 2.1 Chemicals

Phosphate buffer solution (PBS pH 7.2), α-tocopherol (certified reference material), curcumin, propyl gallate (purity ≥98 %), sodium dodecyl sulfate (purity ≥99 %) were all purchased from Sigma-Aldrich (Saint Quentin Fallavier, France). Gallic acid was purchased from Sigma (Saint Quentin Fallavier, France). n-octyl gallate (purity ≥98 %) was purchased from Alfa Aesar. Ethanol and acetone were HPLC grade solvents and purchased from Sigma-Aldrich (Saint Quentin Fallavier, France).

### 2.2 Samples preparation

Stock solutions of gallic acid, propyl gallate, octyl gallate, and α-tocopherol were prepared at concentrations of 200 mM and 20 mM in ethanol, while curcumin was dissolved at 50 mM and 5 mM in acetone. Sodium dodecyl sulfate (SDS) solutions were prepared at two concentrations - 1 mM and 20 mM - in phosphate buffer (pH 7.2). For each experimental condition, the SDS solution was mixed with the antioxidant stock solution to achieve the desired final concentrations in a total volume of 3 mL. Using the 200 mM ethanol-based stock solutions of gallic acid, propyl gallate, octyl gallate, and α-tocopherol, final concentrations of 1, 2.5, 5, and 10 mM were prepared. The corresponding volumes of ethanol added ranged from 15 μL to 150 μL. Alternatively, the 20 mM stock solutions of these antioxidants were used to prepare lower final concentrations of 0.1 and 0.5 mM, with ethanol volumes ranging from 15 μL to 75 μL. Curcumin was diluted from a 50 mM stock solution in acetone to obtain final concentrations of 0.5, 1, 2.5, and 5 mM, requiring 30 μL–300 μL of acetone. For the lowest concentration (0.1 mM), the 5 mM stock solution was used, with 60 μL of acetone added. The samples were incubated at 25 °C with an open cap under gentle orbital stirring (150 rpm) for 3 h using an IKA KS 4000 i-control incubator (IKA, Staufen, Germany). The samples were then centrifuged at 5000 rpm for 5 min (Rotina 380R, Hettich, Westphalie, Germany). Subsequently, 1.5 mL of the supernatant was collected and transferred into a SD-TDA vial, except for α-tocopherol, where the lower phase was collected.

### 2.3 Size distribution - Taylor dispersion analysis (SD-TDA)

The particle size distribution of samples was measured by Size Distribution – Taylor Dispersion Analysis (SD-TDA) using TaylorSizer (Nanoscale Metrix, France). SDS solution with corresponding concentration (either 1 mM or 20 mM) in phosphate buffer (pH 7.2) was used for both conditioning and mobilization steps. The capillary (fused silica tubing), measuring 60 cm with 49.5 cm effective lenght and an internal diameter of 50 μm, was lowered into the vial for sample injection and mobilization. It is initially conditioned with the SDS-buffer solution at 1000 mbar for 120 s. The sample was then injected into the capillary at 25 mbar for 15 s ensuring that the injection is equivalent to 1 % of the total volume of the capillary. Subsequently, the SDS-buffer solution was used for sample mobilization at pressures of 20, 50, 100, 200 or 400 mbar for durations of 2500, 1000, 520, 260, or 130 s, respectively. This process allowed the sample to migrate through the capillary until it was detected by the UV detector, which was set to the wavelengths corresponding to the maximum absorptions of the antioxidants. Finally, the capillary was rinsed with the buffer solution at 1000 mbar for 40 s. All experiments were performed at 25 °C. The resulting peak was analyzed to determine the size distribution using TaylorSizer software (Taylorsoft) developed using a patented algorithm (Cottet et al., 2012). Finally, the hydrodynamic diameter (Dh, nm) of free gallic acid, propyl gallate, and octyl gallate was measured with the molecules dissolved in phosphate buffer (pH 7), whereas curcumin was measured in a phosphate buffer (pH 7)/acetone mixture (80/20, v/v), and α-tocopherol in ethanol. Under these conditions, the sample mobilization solution was matched to the solvents used for antioxidant preparation.

### 2.4 Quantification of antioxidant loading and partitioning based on Taylorgram area integration

Antioxidant partitioning was assessed using Size Distribution - Taylor Dispersion Analysis (SD-TDA) with a UV-TaylorSizer instrument (Nanoscale Metrix, France). The analysis was selectively adjusted to optimize the Taylorgram detection at the maximum specific absorbance wavelength for each antioxidant. The Taylorsizer is able to detect UV–Vis absorbance in the range of 190–1010 nm. Specifically, gallic acid, propyl gallate, and octyl gallate were measured at 262 nm, curcumin at 424 nm, and α-tocopherol at 292 nm. Additionally, measurements at 207 nm were recorded for all antioxidants to provide complementary information. For each antioxidant, theoretical Taylorgram areas as a function of concentration were established, resulting in a linear calibration curve (y = ax + b), in accordance with the Beer-Lambert law. Antioxidant partitioning, as well as the percentage of loaded antioxidant within different populations, was determined by comparing the integrated experimental Taylorgram areas with the theoretical areas predicted from the calibration curves.

### 2.5 Dynamic light scattering (DLS)

The particle size distribution of samples was measured by Dynamic Light Scattering (DLS) using Zetasizer pro (Malvern Panalytical, UK). A 1.1 mL aliquot of sample was placed into a polystyrene cuvette (Fisherbrand, Fisher Scientific, France). The cuvette was inserted into the measurement chamber and a 1-min stabilization period was allowed prior to measurement. The refractive index was set to 1.505 for the α-tocopherol and 1.33 for dispersant (PBS). The general-purpose analysis model was applied for the measurements performed at 25 °C in triplicate and results were reported as Z-average size (nm) and polydispersity index (PDI), as well as Number (nm) and volume (nm) at the apex of the monomodal distribution.

### 2.6 Statistical analyses

Each sample was formulated in triplicate and the measurement were performed three times on each of the triplicates. The results are presented as mean ± SD. Statistical significance was determined by Student *t*-test using XLSTAT. Values with asterisk are significantly different (p < 0.05).

## 3 Results and discussion

### 3.1 Optimization of experimental conditions in size distribution - Taylor dispersion analysis (SD-TDA): balancing analysis time and sensitivity in size distribution measurements

**Fig. 1:**
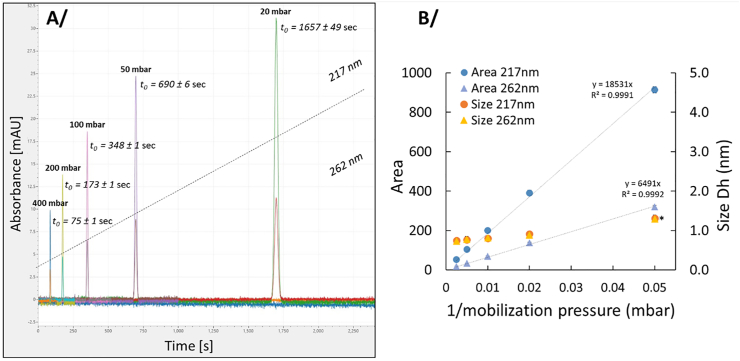
**A/**Taylorgrams at 217 nm and 262 nm of gallic acid (1 mM) with different mobilization pressures: 20, 50, 100, 200 and 400 mbars. Average time ± SD (t_0_) of three independent analyses. **B/**Taylorgram area integration peaks at 217 nm and 262 nm and size hydrodynamic diameter (Dh, nm) of gallic acid (1 mM) with different mobilization pressures, namely 20, 50, 100, 200 and 400 mbars. Size values marked with an asterisk have p-values greater than the significance level (α = 0.05) and are considered statistically significant (n = 3).

Prior to conducting these experiments, it is essential to select the optimal balance between analysis duration and measurement sensitivity. To investigate this, the effect of pressure on the average elution time (t_0_, corresponding to the maximum absorbance position of the elution peak obtained by SD-TDA) and on the hydrodynamic diameter (D_h_, nm) measurement of gallic acid (1 mM) was studied using SD-TDA at a constant injection volume (Fig. 1-A).

Mobilization pressures of 20, 50, 100, 200, and 400 mbar were tested using the same capillary (50 μm × 60 cm bare fused silica). The results showed that lower pressures led to higher t_0_ values, consistent with previous findings (Malburet et al., 2023), indicating that decreasing pressure significantly extends analysis time, which may be a concern in the context of analyzing antioxidant partitioning concentration. The relationship between the integrated area of the Taylorgram peak at 217 nm and 262 nm and the mobilization pressure is presented in Fig. 1-B. Sensitivity increased as pressure decreased, with sensitivity inversely and linearly proportional to pressure, regardless of the selected wavelength. Thus, while lower pressure extends analysis time, it enhances sensitivity and may lower the detection limit. As shown in Fig. 1-B, the hydrodynamic diameter at the lowest pressure (20 mbar) differed slightly, but was statistically significant, from the others. This could be due to slight variations in viscosity at low pressure rates. Thus, considering analysis duration, sensitivity, and measurement accuracy, a pressure of 50 mbar was chosen. This setting allowed automated sample acquisition every 700 s, with stable measurements and improved reproducibility of D_h_ values.

### 3.2 Application of SD-TDA for the evaluation of the hydrodynamic size of gallic acid (GA), propyl gallate (G3) and octyl gallates (G8) in the presence of SDS below (1 mM) and above (20 mM) its critical micelle concentration (CMC)

**Fig. 2:**
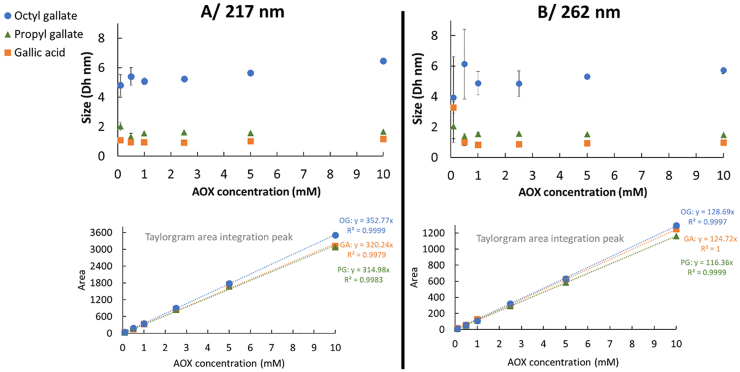
Hydrodynamic diameters (Dh, nm) and integrated Taylorgram peak areas of gallic acid, propyl gallate, and octyl gallate at varying concentrations (0.1–10 mM) in 20 mM SDS micelles ([SDS] > CMC) at 217 nm (A) and 262 nm (B). The mobilization pressure was set at 50 mbar, and analyses were performed in triplicate at 25 °C.

Gallic acid and its gallate derivatives, widely used as antioxidants in the food industry were selected for this initial study aiming to use SD-TDA to measure both the size and partitioning of antioxidants in native-state micelles. The size (Dh, nm) of free gallic acid (GA), propyl gallate (G3) and octyl gallates (G8) in phosphate buffer (pH 7) was analyzed by SD-TDA at 0.95 ± 0.03, 0.98 ± 0.03 and 1.61 ± 0.13 nm, respectively. Fig. 2 shows the hydrodynamic diameter (Dh, nm) and Taylorgram integration peak areas of GA, G3 and G8 at various concentrations (0.1–10 mM) in the presence of 20 mM SDS micelles (above the CMC of SDS), analyzed at 217 nm (A) and 262 nm (B).

Under these conditions, the SD-TDA size distribution analysis only shows a single population, indicating that GA, G3, and G8 do not partition between the continuous phase and SDS micelles. GA and G3 appear to be entirely located in the continuous aqueous phase, as indicated by Dh values which are consistent with their molecular sizes: 1.01 ± 0.11 nm for GA (217 nm) and 1.55 ± 0.16 nm for G3 (217 nm). A slight increase in the hydrodynamic diameter of G3 (from 0.98 ± 0.03 to 1.55 ± 0.16) was observed, potentially due to its partial accumulation in the Stern and/or Gouy-Chapman layers of the micelles (Garcia and Sanz-Medel, 1986). This suggests that G3 is located in the outer region, near the micelle surface, resulting in a modest decrease in its average diffusion coefficient. This observation is consistent with the localization of G3 in SDS micelles as determined by ^1^H NMR (Heins et al., 2007b) and supports the hypothesis that alkyl chain longer than C3 are likely required for significant accumulation within the palisade layer of the micelle (Stöckmann et al., 2000). In contrast, G8 appears to preferentially concentrate into the SDS micelle core, as evidenced by an increase in the size of G8-SDS micelles with G8 concentration, from 4.8 ± 0.7 nm at 0.1 mM to 6.5 ± 0.1 nm at 10 mM (217 nm). Previous studies have similarly reported G8's ability to penetrate more deeply into micellar structures (Heins et al., 2007a). It is also worth noting that although not statistically significant at the tested concentrations, size determinations at 262 nm exhibited greater variability in Dh at the lowest concentration (<0.5 mM), likely due to a reduced signal-to-background ratio.

**Fig. 3:**
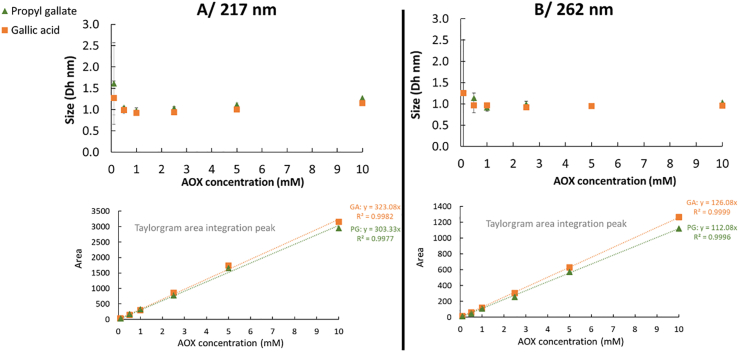
Hydrodynamic diameter (Dh, nm) and integrated Taylorgram peak areas of gallic acid and propyl gallate at varying concentrations (0.1–10 mM) in 1 mM SDS ([SDS] < CMC) at 217 nm (A) and 262 nm (B). The mobilization pressure was set to 50 mbar, and analyses were performed in triplicate at 25 °C.

**Fig. 4:**
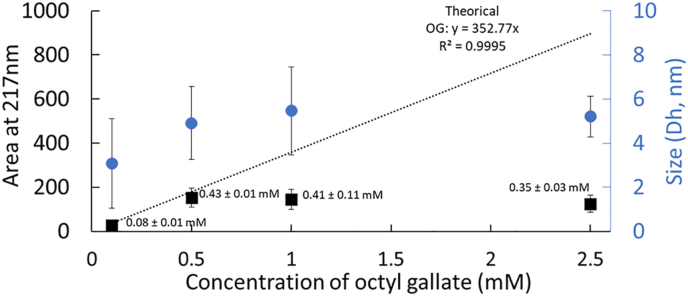
Integrated Taylorgram peak areas at 217 nm and hydrodynamic diameter (Dh, nm) measurements of octyl gallate at varying concentrations (0.1–2.5 mM) in 1 mM SDS (below its CMC). The mobilization pressure was set to 50 mbar, and analyses were performed in triplicate at 25 °C. The concentration of octyl gallate in SDS co-micelles was calculated based on the theoretical Taylorgram area integration value.

In the absence of SDS micelles (1 mM, [SDS] < CMC), SD-TDA size distribution analysis (Fig. 3) revealed a single population for GA and G3, with hydrodynamic diameters comparable to those of the free antioxidant molecules: 1.04 ± 0.19 nm for GA and 1.09 ± 0.11 nm for G3 (217 nm). These results, supported by both the size measurements and the integrated Taylorgram peak area, confirmed that GA remains exclusively locate in the aqueous phase, across all tested concentrations, and is not unaffected by the presence of SDS (even in the presence of SDS micelles, Fig. 2). In contrast, G3 also remains in the aqueous phase but shows sign of interaction with the micelle surface layers, when present. G8 exhibits a distinct behavior due to its low aqueous solubility, rendering it nearly insoluble in the absence of micelles (Fig. 4). Nonetheless, at low concentrations (100 μM–2.5 mM), a population with an average hydrodynamic diameter of approximately 4.7 nm ± 1.7 nm was detected. Estimation of G8 concentration within this population (via comparison of Taylorgram peak areas to the theoretical value) suggests that up to 400 μM of G8 can co-associate in the 1 mM SDS aqueous solution. This observation points to potential self- or co-micellization of G8 with SDS, consistent with the reported CMC of G8 at approximately 50 μM (Maldonado et al., 2011). Intriguingly, this population disappears at higher G8 concentrations, which may be attributed to rapid physical precipitation upon direct mixing with water at excessively high concentrations, rather than to gradual co-association enabled by controlled ethanol evaporation.

α-Tocopherol and curcumin are hydrophobic bioactive compounds whose behavior in micellar systems is of considerable interest to the food industry. Understanding their interactions with these systems is essential for optimizing their stability, bioavailability, and functionality. Moreover, they are expected to exhibit contrasting behaviors in aqueous micellar environments: α-tocopherol tends to form oil droplets upon oversaturation, whereas curcumin crystallizes and precipitates once the saturation capacity of host micelles is exceeded (Song et al., 2023).

### 3.3 Application of SD-TDA to evaluate the size of curcumin in the presence of SDS below (1 mM) and above (20 mM) its CMC

**Fig. 5:**
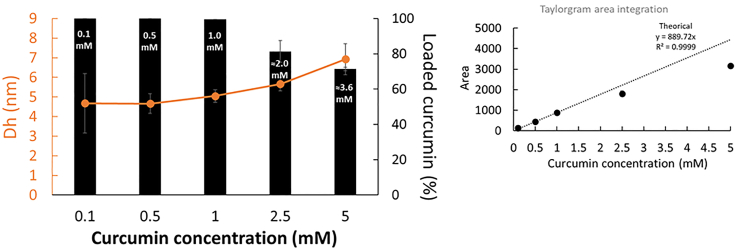
Integrated Taylorgram peak areas at 424 nm and hydrodynamic diameter (D_h_, nm) measurements of curcumin at varying concentrations (0.1–5 mM) in 20 mM SDS micelles (above its CMC). The mobilization pressure was set to 50 mbar, and analyses were performed in triplicate at 25 °C. The concentration (mM) of curcumin in SDS micelles and the loading percentage (%) were calculated based on the added concentration and the theoretical Taylorgram area integration value.

The size (Dh, nm) of free curcumin in phosphate buffer (pH 7)/acetone (80/20, v/v) was analyzed by SD-TDA and found to be 0.97 ± 0.20 nm (424 nm). Fig. 5 shows the hydrodynamic diameter (Dh, nm) and integrated Taylorgram peak areas of curcumin at varying concentrations (0.1–5 mM) in the presence of 20 mM SDS micelles (above its CMC), analyzed at 424 nm.

The SD-TDA size distribution analysis showed a single population, indicating that curcumin does not partition between the continuous phase and SDS micelles but is fully incorporated within the SDS micelles. Notably, the size of curcumin-SDS micelles increased with curcumin concentration, ranging from 4.78 ± 1.5 nm at 0.1 mM to 6.92 ± 0.79 nm at 5 mM. Interestingly, the Taylorgram area integration showed that SDS micelles reached their maximum curcumin loading at 81.3 % when curcumin was added at 2.5 mM (2.03 ± 0.15 mM of curcumin) and 71.4 % at 5 mM (3.57 ± 0.05 mM of curcumin). This suggests that at maximum loading capacity, the curcumin-to-SDS molar ratio was approximately 1:5.5.

**Fig. 6:**
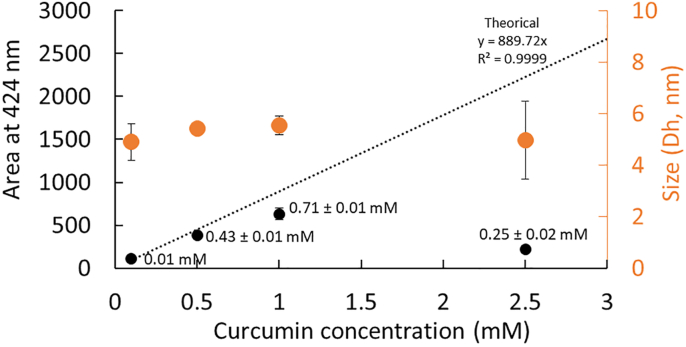
Integrated Taylorgram peak areas at 424 nm and hydrodynamic diameter (Dh, nm) measurements of curcumin at varying concentrations (0.1–2.5 mM) in 1 mM SDS (below its CMC). The mobilization pressure was set to 50 mbar, and analyses were performed in triplicate at 25 °C. The concentration of curcumin in SDS co-micelles were calculated based on the theoretical Taylorgram area integration value.

Interestingly, in the absence of SDS micelles (1 mM, < CMC), the SD-TDA size distribution analysis (Fig. 6) revealed a single population at the lowest concentrations of curcumin (<2.5 mM), with hydrodynamic diameters of approximatively 5.2 nm ± 0.3 nm (424 nm). This result closely resembles previous observations with G8. By plotting the Taylorgram area integration at 424 nm against the theoretical value, the curcumin concentration in this population was estimated, revealing that up to 0.71 ± 0.01 mM of curcumin may co-associate in the 1 mM SDS aqueous solution. This suggests a potential self- or co-micellization of curcumin with SDS. At higher concentrations, this population was no longer observed, further supporting the earlier observation with G8 and suggesting that gradual co-association at appropriate concentrations is necessary to enable the co-micellization process.

### 3.4 Application of SD-TDA to evaluate the size of α-tocopherol-containing assemblies in the presence of SDS above its CMC (20 mM)

**Fig. 7:**
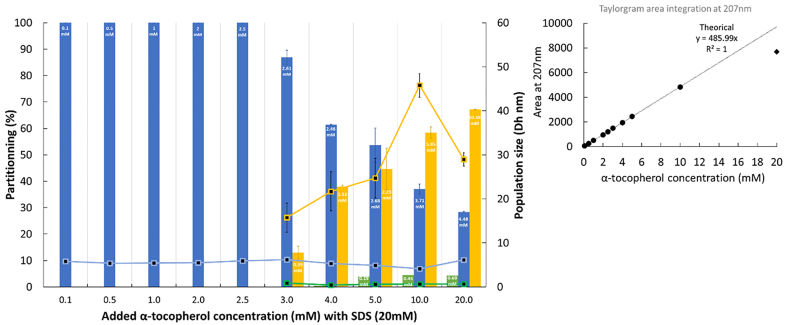
Integrated Taylorgram peak areas at 207 nm, hydrodynamic diameter (D_h_, nm) measurements, and partitioning of α-tocopherol at varying concentrations (0.1–20 mM) in 20 mM SDS micelles (above its CMC). The mobilization pressure was set to 50 mbar, and analyses were performed in triplicate at 25 °C. The concentrations of α-tocopherol in the different populations (free, small micelles and swollen micelles) were calculated based on the added concentration and the theoretical Taylorgram area integration value.

The final assay was conducted with α-tocopherol. Its size (Dh, nm) was analyzed by SD-TDA and found to be at 1.11 ± 0.01 nm (analyzed at 207 nm). Fig. 7 presents the hydrodynamic diameter (Dh, nm) and integrated Taylorgram peak areas of α-tocopherol at varying concentrations (0.1–20 mM) in the presence of 20 mM SDS micelles (above its CMC), analyzed at 207 nm.

SD-TDA size distribution analysis showed a single population when α-tocopherol concentration was ≤2.5 mM, indicating that up to this threshold, α-tocopherol does not partition between the continuous phase and SDS micelles. Under these conditions, the size of α-tocopherol-SDS micelles remains constant, ranging from 5.8 ± 0.4 nm at 0.1 mM to 5.9 ± 0.1 nm at 2.5 mM. However, at concentrations exceeding 2.5 mM, the SD-TDA size distribution revealed at least three distinct populations: free α-tocopherol in the aqueous phase (Dh ∼0.7 ± 0.1 nm), α-tocopherol in small micelles, and a larger population attributed to swollen SDS micelles (Supplementary Fig. S1). The size of the small α-tocopherol-SDS micelles remained relatively unchanged, ranging from 6.2 ± 0.2 nm at 3 mM to 6.3 ± 0.3 nm at 20 mM. In contrast, the size of the swollen micelles increased with α-tocopherol concentration, from 15.8 ± 3.3 nm at 3 mM to 29.0 ± 1.6 nm at 20 mM, with a maximum observed at 10 mM (45.8 ± 2.7 nm). Taylorgram area integration showed that at α-tocopherol concentrations below 10 mM, the entire added amount was incorporated into the 20 mM SDS micelles. However, at 20 mM, approximately 4.3 mM (∼21 %) of α-tocopherol remained insoluble and was removed as a lipid-rich upper phase following centrifugation. Up to 2.5 mM, 100 % of α-tocopherol was incorporated into small micelles. Based on observations at higher concentrations, one may consider that free α-tocopherol may be present at such lower concentrations, but it remains undetectable, falling below the limit of detection. From 3 mM to 20 mM, α-tocopherol was distributed among free molecules (from 0.12 ± 0.25 % to 4.4 ± 0.1 %), small micelles (from 86.9 ± 2.7 % to 28.4 ± 0.1 %), and swollen micelles (from 13.0 ± 2.4 % to 67.2 ± 0.2 %). Concentration analysis across these populations indicates that at maximum loading capacity, the α-tocopherol-to-SDS molar ratio is approximately 1:7 in small micelles. Beyond this point, α-tocopherol is progressively redistributed from small to swollen micelles, with a steady, concentration-dependent increase in the amount of α-tocopherol within swollen micelles, reaching approximately 10.6 ± 0.1 mM. These findings suggest that the partitioning of α-tocopherol between different micellar environments is highly concentration-dependent.

### 3.5 Assessing the effectiveness of SD-TDA versus DLS in analyzing small and/or polydisperse particle size distributions

Microscopy techniques, such as transmission electron microscopy (TEM) and cryogenic TEM (cryo-TEM), are highly effective for analyzing the size distribution and morphology of nano-objects. However, these methods are costly, labor-intensive, and often face limitations in resolving very small nanostructures. As alternatives, scattering and diffraction-based techniques, such as nanoparticle tracking analysis (NTA) and dynamic light scattering (DLS), are widely used to estimate the size distribution of micelles, lipid assemblies (e.g., liposomes), and oil droplets in emulsions. Nevertheless, these methods are limited in their ability to accurately analyze sub-10 nm objects as such small particles scatter light weakly and may not be efficiently detected. Additionally, the presence of larger particles in a sample can interfere with the accurate detection and sizing of smaller species. Table 1 presents DLS measurements of α-tocopherol at concentrations ranging from 2 to 20 mM in 20 mM SDS micelles (above the CMC). In the absence of swollen micelles, number and volume-weighted DLS distributions successfully identified a population of small-sized population (∼5 nm), while intensity-weighted distributions failed to provide a reliable estimation. As expected, at concentrations above 3 mM - where swollen micelles begin to form - DLS yielded a primarily monodisperse distribution (albeit with minor heterogeneity) centered around the size of these larger aggregates. Furthermore, DLS was not able to resolve multiple populations or provide evidence of progressive size increases. In contrast, SD-TDA provides a mass-weighted size distribution, which is compatible with mass concentration-sensitive detectors (Cottet et al., 2007). DLS, by comparison yields an intensity-weighted distribution which disproportionately favor larger particles. This fundamental difference explains why the DLS size distribution is broader than that obtained by TDA. These findings are consistent with previous studies, where DLS measurement of micellar solutions containing α-tocopherol concentrations above 2 mM (in polyoxyethylene ethers, e.g. Brij) revealed only a single population corresponding to α-tocopherol-rich oil droplets (Song et al., 2023).

**Table 1:** Formatting dynamic light scattering (DLS) analysis of α-tocopherol at varying concentrations (2–20 mM) in 20 mM SDS micelles (above the CMC). Analyses were performed in triplicate at 25 °C and particle size distributions (nm) are reported based on number, volume and intensity-weighted analyses.

Concentration of α-tocopherol	Number	Volume	Intensity-based distribution	
	Peak of the monomodal distribution (nm)		Z-average (nm)	Polydispersity index (PI)
**2 mM**	5.3 ± 0.0	5.6 ± 0.5	–	–
**3 mM**	36.3 ± 8.0	44.0 ± 6.6	68.48 ± 0.90	0.21 ± 0.01
**4 mM**	32.6 ± 8.5	44.0 ± 6.6	69.30 ± 1.78	0.22 ± 0.03
**5 mM**	27.8 ± 0.0	35.8 ± 3.0	65.02 ± 0.63	0.19 ± 0.01
**10 mM**	30.1 ± 8.7	36.3 ± 8.0	74.98 ± 0.01	0.19 ± 0.01
**20 mM**	27.9 ± 4.2	32.5 ± 4.9	93.59 ± 0.08	0.29 ± 0.08

## 4 Conclusion

Size Distribution - Taylor Dispersion Analysis (SD-TDA) revealed distinct partitioning behaviors of antioxidants in SDS micellar systems, underscoring its value for investigating native-state micellar interactions. Gallic acid (GA) and propyl gallate (G3) were found to predominantly reside in the aqueous phase, as evidenced by hydrodynamic diameters (Dh) that closely matched their molecular sizes (1.01 ± 0.11 nm for GA and 1.55 ± 0.16 nm for G3). However, the slight increase in G3's Dh suggests partial association with micellar interfacial regions, such as the Stern or Gouy-Chapman layers. In contrast, octyl gallate (G8) preferentially partitioned into SDS micelle, with the micellar size increasing from 4.8 ± 0.7 nm to 6.5 ± 0.1 nm at 10 mM G8. Similarly, curcumin was fully solubilized within SDS micelles, with Dh values increasing from 4.68 ± 1.52 nm at the lowest curcumin concentration to 6.92 ± 0.79 nm at maximal loading capacity. Below the CMC, both curcumin and G8 displayed populations with Dh values in the range of ∼4.7–5.2 nm, indicating potential self- or co-micellization with SDS molecules. α-Tocopherol was also predominantly localized within SDS micelles; however, at concentrations exceeding 2.5 mM, SD-TDA distinguished three co-existing populations: free α-tocopherol in the aqueous phase (∼0.7 nm), α-Tocopherol in small micelles (∼6.2–6.3 nm), and a larger population attributed to swollen micelles, which increased in size up to 45.8 ± 2.7 nm at 10 mM of added α-tocopherol. Unlike DLS, TDA provided high-resolution differentiation of coexisting populations and accurate quantification of micellar partitioning behavior. These findings highlight the potential of SD-TDA as a powerful tool for elucidating antioxidant–surfactant interactions and in assessing antioxidant partitioning in micelles, insights that are essential for optimizing antioxidant efficacy in emulsified formulations, particularly in the context of lipid oxidation.

## CRediT authorship contribution statement

**Erwann Durand:** Conceptualization, Methodology, Investigation, Funding acquisition, Formal analysis, Supervision, Validation, Writing – original draft, Writing – review & editing. **Théo Troncho:** Methodology, Investigation, Formal analysis, Validation, Writing – original draft, Writing – review & editing. **Pierre Villeneuve:** Methodology, Investigation, Formal analysis, Validation, Writing – original draft, Writing – review & editing.

## Funding sources

This work was supported by the French government under the France 2030 initiative, managed by the National Research Agency (ANR), under grant number “ANR-23-DIVP-0003”, project “M2ProLIV”.

## Declaration of competing interest

The authors declare that they have no known competing financial interests or personal relationships that could have appeared to influence the work reported in this paper.

## Data Availability

Data will be made available on request.

## References

[bib1] Am A., Trapiella-Alfonso L., Izabelle C., Saubamea B., Doan B.T., d'Orlyé F., Varenne A. (2024). Amphiphilic self-assembling peptides: formulation and elucidation of functional nanostructures for imaging and smart drug delivery: amphiphilic self-assembling peptides: formulation and elucidation of functional nanostructures for imaging and smart drug delivery: a. Anal. Bioanal. Chem..

[bib2] Balog S., Urban D.A., Milosevic A.M., Crippa F., Rothen-Rutishauser B., Petri-Fink A. (2017). Taylor dispersion of nanoparticles. J. Nanoparticle Res..

[bib3] Barden L., Decker E.A. (2013). Lipid oxidation in low-moisture food: a review. Crit. Rev. Food Sci. Nutr..

[bib4] Cottet H., Biron J.P., Martin M. (2007). Taylor dispersion analysis of mixtures. Anal. Chem..

[bib5] Cottet H., Cipelletti L., Martin M., Biron J.-P. (2012). WO2014001409 A1.

[bib6] Domínguez R., Pateiro M., Gagaoua M., Barba F.J., Zhang W., Lorenzo J.M. (2019). A comprehensive review on lipid oxidation in meat and meat products. Antioxidants.

[bib7] Durand E., Laguerre M., Bourlieu-Lacanal C., Lecomte J., Villeneuve P. (2025). Navigating the complexity of lipid oxidation and antioxidation: a review of evaluation methods and emerging approaches. Prog. Lipid Res..

[bib8] Fortunatus R.M., Balog S., Sousa F., Vanhecke D., Rothen-Rutishauser B., Taladriz-Blanco P., Petri-Fink A. (2025). Taylor dispersion analysis and release studies of β-carotene-loaded PLGA nanoparticles and liposomes in simulated gastrointestinal fluids. RSC Adv..

[bib9] Franzen U., Vermehren C., Jensen H., Østergaard J. (2011). Physicochemical characterization of a PEGylated liposomal drug formulation using capillary electrophoresis. Electrophoresis.

[bib10] Garcia M.E.D., Sanz-Medel A. (1986). Dye-surfactant interactions: a review. Talanta.

[bib11] German J.B. (1999). Adv Exp Med Biol.

[bib12] Hawe A., Hulse W.L., Jiskoot W., Forbes R.T. (2011). Taylor dispersion analysis compared to dynamic light scattering for the size analysis of therapeutic peptides and proteins and their aggregates. Pharm. Res..

[bib13] Heins A., McPhail D.B., Sokolowski T., Stöckmann H., Schwarz K. (2007). The location of phenolic antioxidants and radicals at interfaces determines their activity. Lipids.

[bib14] Heins A., Sokolowski T., Stöckmann H., Schwarz K. (2007). Investigating the location of propyl gallate at surfaces and its chemical microenvironment by (1)H NMR. Lipids.

[bib15] Hennebelle M., Villeneuve P., Durand E., Lecomte J., van Duynhoven J., Meynier A., Yesiltas B., Jacobsen C., Berton-Carabin C. (2024). Lipid oxidation in emulsions: new insights from the past two decades. Prog. Lipid Res..

[bib16] Huang S.W., Frankel E.N., Aeschbach R., German J.B. (1997). Partition of selected antioxidants in corn oil−water model systems. J. Agric. Food Chem..

[bib17] Inchingolo R., Bayram I., Uluata S., Kiralan S.S., Rodriguez-Estrada M.T., McClements D.J., Decker E.A. (2021). Ability of sodium dodecyl sulfate (SDS) micelles to increase the antioxidant activity of α-Tocopherol. J. Agric. Food Chem..

[bib18] Kiralan S.S., Doʇu-Baykut E., Kittipongpittaya K., McClements D.J., Decker E.A. (2014). Increased antioxidant efficacy of tocopherols by surfactant solubilization in oil-in-water emulsions. J. Agric. Food Chem..

[bib19] Malburet C., Leclercq L., Cotte J.F., Thiebaud J., Bazin E., Garinot M., Cottet H. (2022). Taylor dispersion analysis to support lipid-nanoparticle formulations for mRNA vaccines. Gene Ther..

[bib20] Malburet C., Leclercq L., Cotte J.F., Thiebaud J., Bazin E., Garinot M., Cottet H. (2022). Size and charge characterization of lipid nanoparticles for mRNA vaccines. Anal. Chem..

[bib21] Malburet C., Martin M., Leclercq L., Cotte J.F., Thiebaud J., Biron J.P., Chamieh J., Cottet H. (2023). Optimization of limit of detection in taylor dispersion analysis: application to the size determination of vaccine antigens. Talanta Open.

[bib22] Maldonado O.S., Lucas R., Comelles F., Jesús González M., Parra J.L., Medina I., Morales J.C. (2011). Synthesis and characterization of phenolic antioxidants with surfactant properties: Glucosyl- and glucuronosyl alkyl gallates. Tetrahedron.

[bib23] McClements D., Decker E. (2000). Lipid oxidation in oil-in-water emulsions: impact of molecular environment on chemical reactions in heterogeneous food systems. J. Food Sci..

[bib24] Moser M.R., Baker C.A. (2021). Taylor dispersion analysis in fused silica capillaries: a tutorial review. Anal. Methods.

[bib25] Norrild R.K., Mason T.O., Boyens-Thiele L., Ray S., Mortensen J.B., Fritsch A.W., Iglesias-Artola J.M., Klausen L.K., Stender E.G.P., Jensen H., Buell A.K. (2024). Taylor dispersion-induced phase separation for the efficient characterisation of protein condensate formation. Angew. Chem., Int. Ed..

[bib26] Obeid S., Chamieh J., Mai T.D., Morani M., Reyre M., Krupova Z., Defrenaix P., Cottet H., Taverna M. (2023). Fast, simple and calibration-free size characterization and quality control of extracellular vesicles using capillary taylor dispersion analysis. J. Chromatogr. A.

[bib27] Roca S., Leclercq L., Biron J.P., Martin M., Cottet H. (2024). Preventing the impact of solute adsorption in taylor dispersion analysis: application to protein and lipid nanoparticle analysis. J. Chromatogr. A.

[bib28] Roca S., Leclercq L., Biron J.P., Martin M., Cottet H. (2024). Preventing the impact of solute adsorption in taylor dispersion analysis: application to protein and lipid nanoparticle analysis. J. Chromatogr. A.

[bib29] Schaich K.M. (2020).

[bib30] Song H.Y., McClements D.J., Choi S.J. (2023). Solubilization of α-tocopherol and curcumin by polyoxyethylene alkyl ether surfactants: effect of alkyl chain structure. Food Chem..

[bib31] Stöckmann H., Schwarz K., Huynh-Ba T. (2000). Influence of various emulsifiers on the partitioning and antioxidant activity of hydroxybenzoic acids and their derivatives in oil-in-water emulsions. JAOCS, Journal of the American Oil Chemists’ Society.

[bib32] Taylor G.I. (1953). Dispersion of soluble matter in solvent flowing slowly through a tube. Proc R Soc Lond A Math Phys Sci.

[bib33] Villeneuve P., Bourlieu-Lacanal C., Durand E., Lecomte J., McClements D.J., Decker E.A. (2023). Lipid oxidation in emulsions and bulk oils: a review of the importance of micelles. Crit. Rev. Food Sci. Nutr..

[bib34] Wang X., Chen Y., McClements D.J., Peng D., Chen H., Xu S., Deng Q., Geng F. (2024). Regulation of microlocalization of antioxidants by surfactant micelles in oil-in-water emulsions. J. Agric. Food Chem..

