# Supplementary material for: Evaluation of antioxidant–surfactant interactions using size distribution Taylor dispersion analysis: measuring antioxidant partitioning and size of native-state micelles
Source: Curr Res Food Sci. 2025 Jul 11;11:101142. doi: 10.1016/j.crfs.2025.101142 (PMC12281138; doi:10.1016/j.crfs.2025.101142)
Supplement: Multimedia component 1 [file mmc1.docx]

**Supplementary information**

**
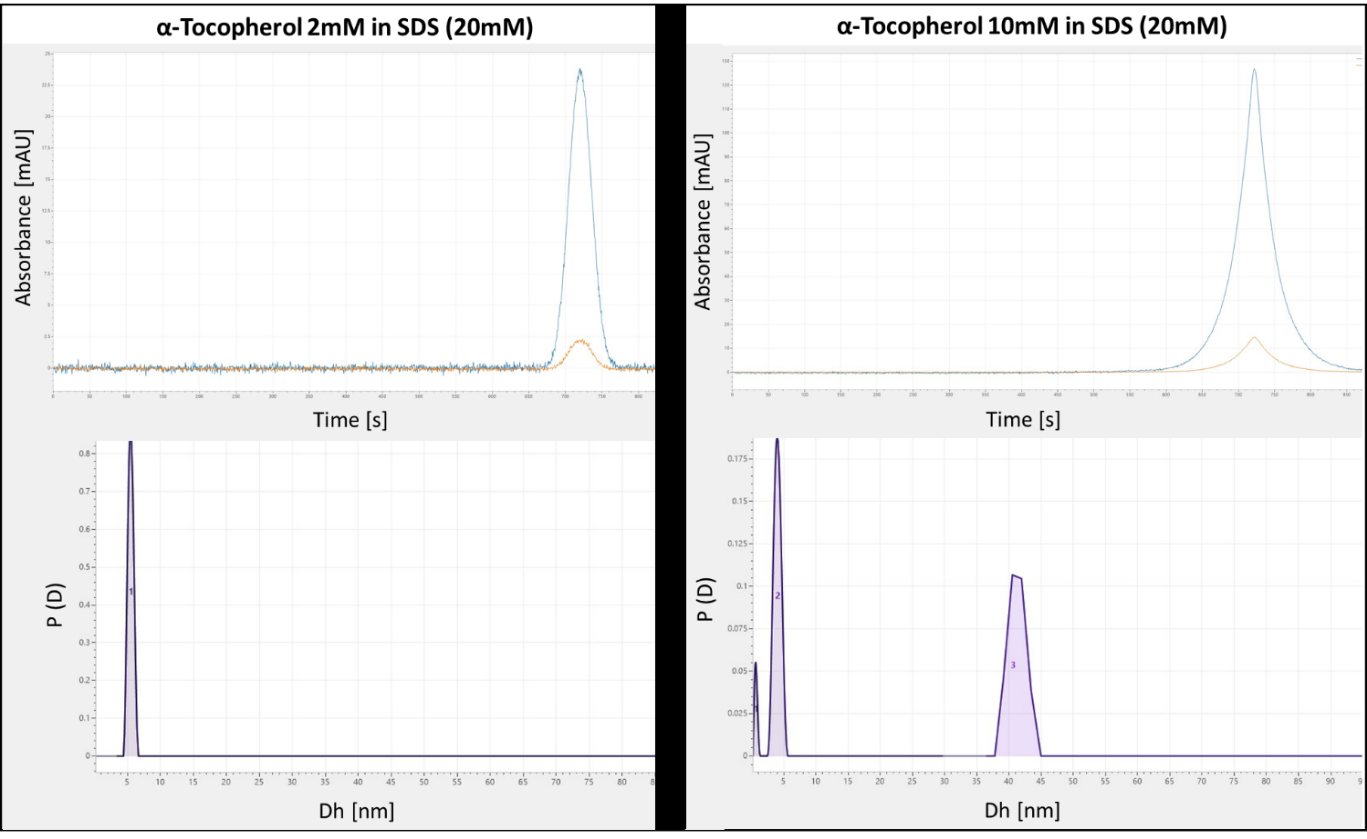
**

**Figure S1:** Representative Taylorgrams at 207 nm and 292 nm, and corresponding hydrodynamic diameters (Dₕ, nm) of α-tocopherol distributed across distinct populations (free, small micelles, and swollen micelles) at two representative concentrations (2 mM and 10 mM) in 20 mM SDS micelles (above the CMC). The mobilization pressure was set to 50 mbar, and analyses were conducted at 25°C.
